# The Influence of Environmental Variables on the Presence of White Sharks, C*archarodon carcharias* at Two Popular Cape Town Bathing Beaches: A Generalized Additive Mixed Model

**DOI:** 10.1371/journal.pone.0068554

**Published:** 2013-07-16

**Authors:** Kay Weltz, Alison A. Kock, Henning Winker, Colin Attwood, Monwabisi Sikweyiya

**Affiliations:** 1 Marine Research Institute and Zoology Department, University of Cape Town, Rondebosch, Cape Town, South Africa; 2 Shark Spotters, Fish Hoek, Cape Town, South Africa; University of California Davis, United States of America

## Abstract

Shark attacks on humans are high profile events which can significantly influence policies related to the coastal zone. A shark warning system in South Africa, *Shark Spotters*, recorded 378 white shark (*Carcharodon carcharias*) sightings at two popular beaches, Fish Hoek and Muizenberg, during 3690 six-hour long spotting shifts, during the months September to May 2006 to 2011. The probabilities of shark sightings were related to environmental variables using Binomial Generalized Additive Mixed Models (GAMMs). Sea surface temperature was significant, with the probability of shark sightings increasing rapidly as SST exceeded 14°C and approached a maximum at 18°C, whereafter it remains high. An 8 times (Muizenberg) and 5 times (Fish Hoek) greater likelihood of sighting a shark was predicted at 18°C than at 14°C. Lunar phase was also significant with a prediction of 1.5 times (Muizenberg) and 4 times (Fish Hoek) greater likelihood of a shark sighting at new moon than at full moon. At Fish Hoek, the probability of sighting a shark was 1.6 times higher during the afternoon shift compared to the morning shift, but no diel effect was found at Muizenberg. A significant increase in the number of shark sightings was identified over the last three years, highlighting the need for ongoing research into shark attack mitigation. These patterns will be incorporated into shark awareness and bather safety campaigns in Cape Town.

## Introduction

The presence of large predatory sharks, such as white sharks (*Carcharodon carcharias*) in inshore coastal waters increases the risk of encounters with people using these areas and may lead to negative interactions, such as shark attacks. By drawing significant media attention, shark attacks negatively impact coastal tourism and public perceptions of sharks, often resulting in local business losses, membership declines in social and life-saving clubs, and a reluctance of the public to support shark conservation campaigns [1], [2], [3]. Politically driven policy actions have traditionally aimed to reduce shark abundance or exclude sharks from popular bathing areas [3]. Such intrusive policies may create conflict with conservation objectives. At a time when shark populations around the world are facing increasing threats from fishing and habitat destruction [4], there is less justification and support for measures which involve the killing of large sharks, especially as the large-scale removal of top-order predators may compromise marine ecosystem functioning with undesirable ecological consequences [5]. As a result, more ecologically responsible approaches are being sought to reduce the risk of shark attack, which include attempts to understand and exploit patterns in shark behaviour to minimise the likelihood of shark encounters [2], [6].

White sharks have been implicated in 346 unprovoked attacks on humans worldwide, of which 102 were fatal since 1839, with a steady increase in the frequency of attacks [7], [2]. Shark attacks in Cape Town, South Africa, have followed this global trend and increased over the last decade with 27 recorded shark attacks since 1960, of which 14 occurred since January 2000 [8], [9]. Furthermore, four attacks in Cape Town between 2000 and 2010 were fatal [8], [9], compelling the local authorities i.e. City of Cape Town, to act by convening a specialist workshop to review existing options to reduce the risk of shark attack [10]. A similar spate of shark attacks occurred in KwaZulu-Natal in the 1940’s and 1950’s, involving white sharks and bull sharks (*Carcharhinus leucas*), which precipitated the use of bather protection gill nets (shark nets) to reduce local shark abundance, and thus the risk of encounters, in the inshore area [11]. These nets act indiscriminately, and reduce the abundance of several chondrichthyan species, many of which are not a threat to humans, as well as causing mortalities of cetaceans and turtles [12].

Following the specialist review, the same strategy was not recommended in Cape Town, because white sharks aggregate there year-round [9], [13] and an attempt to reduce their abundance in this way could have a detrimental effect on their population [10], [2]. White sharks are a threatened species, classified as ‘Vulnerable’ on the IUCN Red List of Threatened Species, with relative low abundances even in aggregation areas [14], [15], and are protected under South African law [16]. Bycatch of cetaceans and other marine life in traditional ‘shark nets’ is expected to be excessive in False Bay, with its diverse and abundant marine life [10]. Increased environmental awareness among the public also mitigates against the use of destructive methods, particularly those that target threatened species. As a result, the recommendations included supporting ongoing research on white shark presence and behaviour at popular beaches and for the City to formally formal adopt a shark warning system called ‘Shark Spotters’ [10]. Most recently, after another two attacks in 2011 and 2012, one of which was fatal, the response has been to trial an exclusion net in a high risk encounter area at one of the local beaches, Fish Hoek [17].

Shark Spotters were introduced along Cape Town’s beaches in 2004, and formally adopted by the City of Cape Town in 2006. This bather safety program employs individuals who spot and record white sharks in the inshore area at seven popular beaches in Cape Town, warning and evacuating water-users when white sharks are present [9]. The programme also incorporates research, education and awareness in their shark safety campaigns [9]. The objective of this study was to use a long-term dataset in white shark sightings recorded by Shark Spotters to identify patterns in the presence of white sharks at these popular bathing sites by investigating the relationship to potential environmental predictor variables, including sea surface temperature (SST), wind speed, time of the day and lunar phase.

## Methods

### Study Area

**Figure 1:**
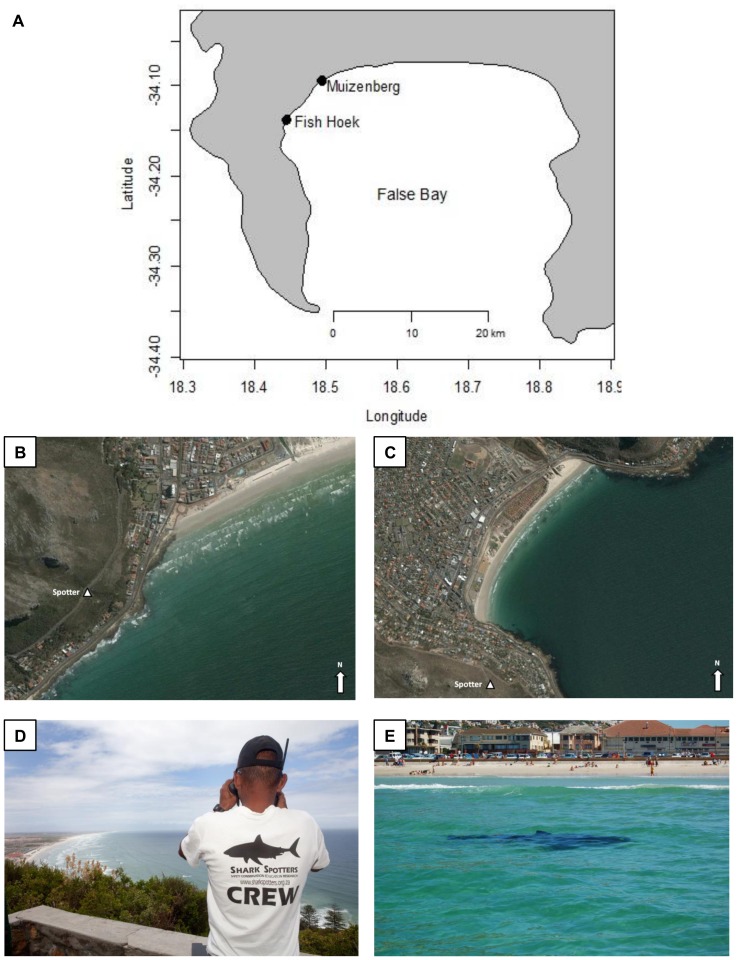
Location of study beaches in False Bay. Map of False Bay (A) illustrating the location of Muizenberg (B) and Fish Hoek (C) where Shark Spotters are positioned strategically above the popular recreational beaches and watch for sharks. Shark Spotters are positioned at an elevated mountain-side position (photo: Muizenberg) (D). White sharks are regularly sighted close inshore at these beaches (photo: Fish Hoek) (E).

Shark sightings were recorded at seven Cape Town beaches by dedicated shark spotters [9]. This study only analysed the data from two of the beaches, namely, Muizenberg (S34°.10970, E18°.4697) and Fish Hoek (S34°.14141, E18°.4330) (Fig. 1). These two beaches were selected because they had significantly higher numbers of shark sightings over the study period which allowed for robust statistical analysis, and are the two most popular recreational swimming and surfing beaches in False Bay with the highest number of human-shark encounters [8], [9]. A summary of the frequency of shark sightings for all beaches is provided in Kock *et al.* (2012).

### Data Collection and Preparation

**Figure 2:**
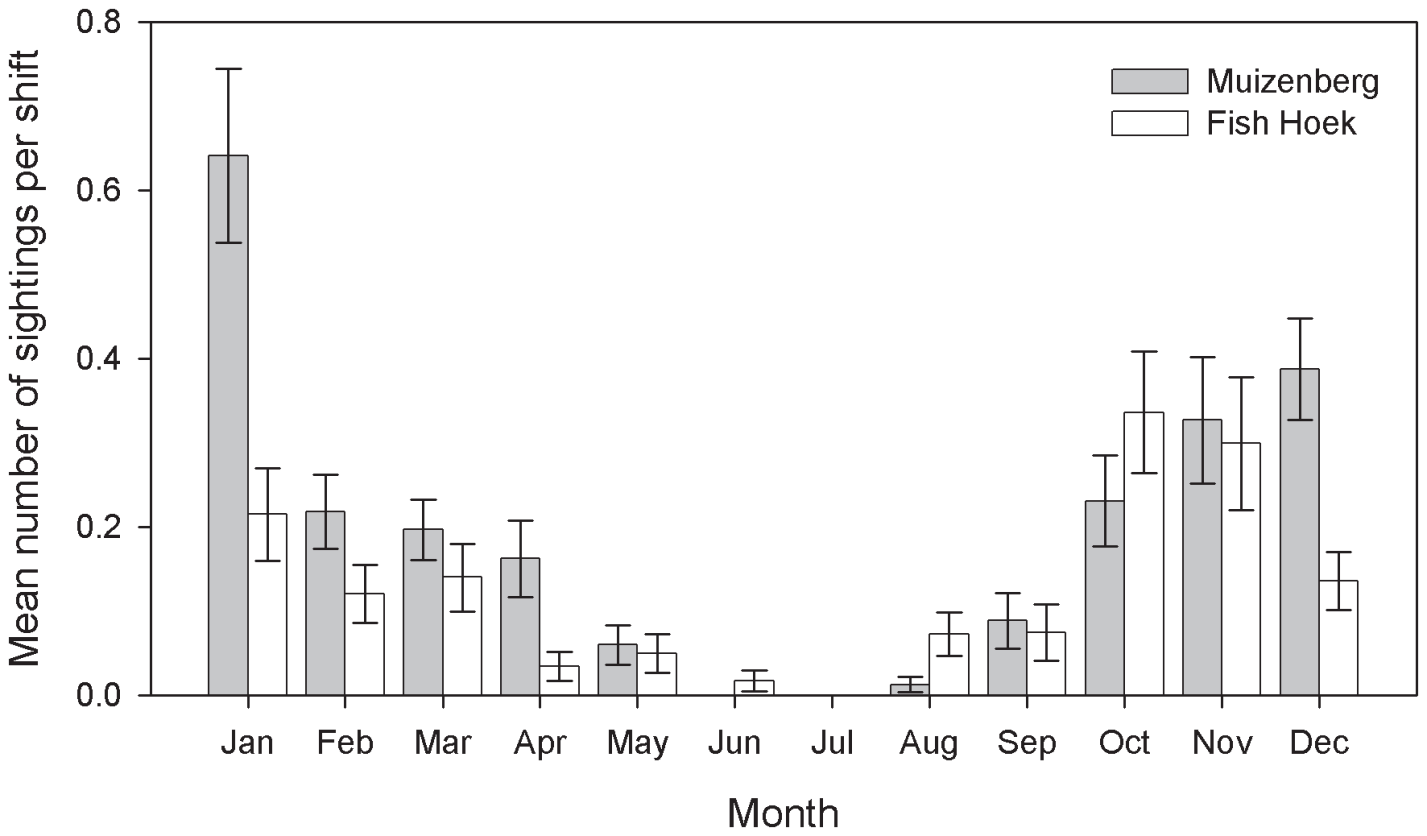
The average number of white shark sightings per spotting shift at Muizenberg and Fish Hoek from January 2006 to June 2011. This study focused on the months September to May, while the months June to August were excluded from this study. Error bars indicate ±1 standard error.

Individual shark spotters were assigned to six-hour long shifts i.e. morning (7∶00–13∶00) and afternoon (13∶00–19∶00) shifts. We restricted our analyses to the months of September to May because white sharks showed a strong seasonal trend in occurrence, with very few sightings between June to August (Fig. 2). The latter is corroborated by a recent study based on acoustic data, which revealed that June to August was associated with the lowest detection rates of white sharks in the inshore region of False Bay [13]. Data from five years were available, i.e. from September to May, for 2006 to 2011. Environmental data were externally sourced for the study period. Daily measurements of sea surface temperature (SST) for Muizenberg and Fish Hoek were provided by the South African Weather Service (SAWS). Those data were available for the study period and were taken with a manual thermometer. Records with missing SST information were excluded from the final datasets. The lunar cycle was grouped according to the eight standard moon phases (1) full moon (2) waxing gibbous (3) first quarter (4) waxing crescent (5) new moon (6) waning crescent (7) first quarter and (8) waning gibbous. Wind speed (ms^−1^) and wind direction measured at Cape Town International Airport was provided by SAWS. Hourly measures of wind speed were averaged for each shift. Wind direction was grouped into the four categories long-, cross-, on- and off-shore direction. The probability of sighting a shark is likely dependent on the abilities and experience of the person spotting. Thus, the identity of individual spotters were assigned to the corresponding morning or afternoon spotting shifts and included as a random effect in the model. The data were filtered to remove all records associated with spotters who had less than 100 recorded shifts; in an attempt to provide consistency between individual spotters and their spotting skills.

### Data Analysis

Shark sightings data included a high proportion of zero sightings and a maximum of four shark sightings per shift. It was not possible to distinguish whether each sighting represented a different shark or if the same shark was recounted as it moved in and out of the field of view. To overcome this issue, we modelled the probability of sighting at least one white shark during a shift as a binomial response. Cleveland dot-plots and box-plots revealed that SST, wind speed, wind direction and lunar phase data showed an adequate spread over the study months, with no true outliers present. Inspections of multi-panel scatterplots indicated no co-linearity between any pair of the environmental variables, thus all variables were retained for the analyses.

Generalized Additive Mixed Models (GAMMs) [18] were used to examine the relationships between the probability of a shark sighting i.e. no sharks sighted per shift (0) and at least one shark sighting per shift (1) and the predictor variables, assuming a binomial error model. The full GAMM, evaluated for each beach independently, included the smoothing functions for the variables ‘SST’, ‘Lunar Phase’, ‘Wind Speed’ and ‘Year’, the categorical variables ‘Wind Direction’ and ‘Shift’ and a random effect for ‘Spotter’, such that:
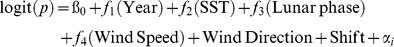
where logit denotes the binomial link function, *p* is the probability of sighting a shark during a shift, *f*_1–4_ denotes the smoothing functions realized by thin plate spline regression functions [18] and *a*_i_ is the random effect for spotter [19]. The reason for treating spotter as a random effect was because of concerns that multiple observations made by the same spotter will cause pseudo-replication, which can subsequently result in overestimated precision and significance levels of the model parameters. The error structure of GAMM corrects for the non-independence of statistical units and permits the ‘random effects’ variance explained at different levels of clustering to be decomposed. The inclusion of individual spotter as a random effect enabled us to account for lack of independence between observations for each Spotter.

The most parsimonious models were selected by first evaluating the random effect for its significance in the full model and then determining the optimal combination of predictor variables using the Akaike’s Information Criterion [19]. Sequential F-tests were used to determine the predictor variables that contributed significantly (p<0.05) to the deviance explained [18]. Finally, the probabilities of sighting a shark were predicted for all significant environmental variables to determine enhanced shark sighting conditions for each beach to increase bather safety. All analyses were conducted using the ‘mgcv’ package [18], which is available in the statistical platform R [20].

## Results

After filtering the data to include only the months September to May, a total of 2022 shifts and 310 shark sightings (Muizenberg) and 1668 shifts and 97 shark sightings (Fish Hoek) were included in the analyses. The overall probabilities of sighting at least one white shark per shift over this period were relatively high with 4.4% for Fish Hoek and 10.0% for Muizenberg.

GAMMs fitted with a random effect for ‘Spotter’ provided a better model for both Fish Hoek and Muizenberg, as judged by the AIC. Consequently, random effects were included in the final GAMMs. Results were similar for both beaches where SST, lunar phase and year were found to be significant (Table 1), but wind speed and wind direction were not significant (p>0.05) and consequently dropped from the models. Differences between morning and afternoon shifts were only found to be significant at Fish Hoek and therefore the variable shift was dropped from the final Muizenberg model.

**Table 1:** Summary statistics for covariates tested in the binomial GAMMs fitted to probabilities of white shark sightings.

	Muizenberg		Fish Hoek	
Covariate	F-test	*p*-value	F-test	*p*-value
s(SST)	16.56	**<0.001**	5.31	**<0.01**
s(Moon )	3.78	**<0.05**	4.67	**<0.01**
s(Year)	12.57	**<0.001**	8.62	**<0.001**
s(Wind Speed)	0.15	0.699	1.85	0.174
Wind Direction	0.71	0.549	0.20	0.895
Shift	0.09	0.760	6.94	**<0.01**

Significant values are in bold.

**Figure 3:**
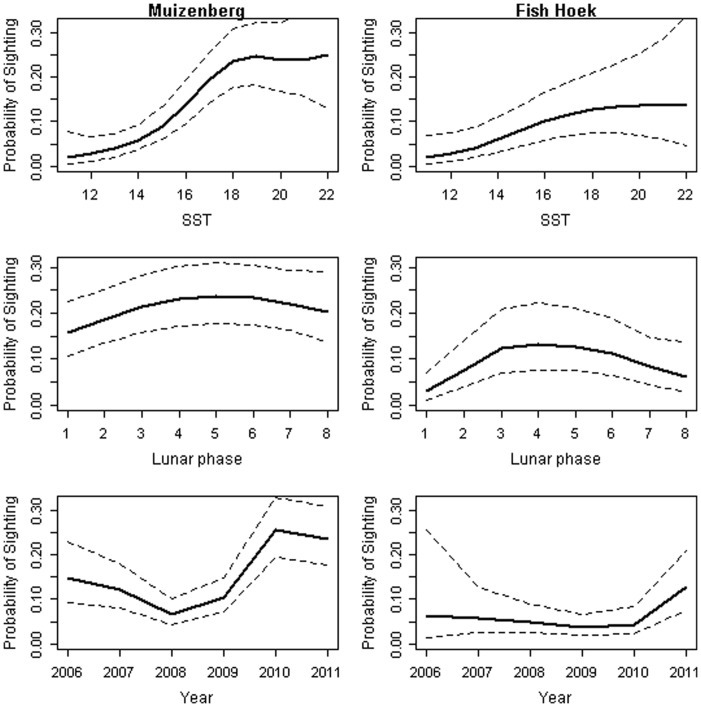
GAMM trends for shark sightings and environmental variables at Muizenberg and Fish Hoek. Trends for the significant variables ‘SST’, ‘Lunar Phase’ and ‘Year’ included in the binomial GAMMs for the beaches Muizenberg (left panel) and Fish Hoek (right panel). Dashed lines represent 95% confidence intervals.

To predict the influence of individual predictor variables on the probability of sighting a white shark, we constructed a reference set of standardized conditions by setting ‘SST’ to 18°C, ‘Lunar phase’ to new moon (phase 5) and ‘Year’ to 2011 [18], [19]. Afternoon shifts were chosen as the standard for predictions based on Fish Hoek data. White shark sightings were recorded over a SST range of 9–22°C at Muizenberg and Fish Hoek. The influence of SST was consistent at both beaches (Fig. 3). The trend revealed that probabilities of shark sightings started to increase rapidly as SST exceeded 14°C and approached a maximum at 18°C, where after it remains high. Based on the reference set of standardized conditions, the probability of sighting a white shark was predicted to be 8 times higher at Muizenberg and 5 times higher at Fish Hoek when the SST was 18°C compared to 14°C. The influence of lunar phase was also significant and consistent for both beaches. Shark sightings increased at new moon and were typically lowest at full moon. At Fish Hoek the probability of sighting a shark was 4 times greater at new moon than at full moon. At Muizenberg the same trend was evident, but to a lesser degree, with the likelihood of sighting a shark increasing 1.5 times at new moon when compared to full moon. Trends for the effect of year indicated an increase in the probability of shark sightings from 2008 onwards for Muizenberg. Sighting probabilities at Fish Hoek showed a slight yet consistent decline until 2009 and increased in 2010 (Fig. 3). At Fish Hoek, the probability of sighting a shark was 1.6 times higher during the afternoon shift compared to the morning shift. No difference was found between morning and afternoon shifts at Muizenberg.

## Discussion

There is a strong seasonal component to the presence of white sharks along the bathing beaches of False Bay, with a peak in occurrence over spring and summer. This is corroborated by Kock *et al*. [13] which demonstrated that peak detection times for white sharks inshore occurred over this period, most likely due to an increase in prey availability. The effects of temperature and season are not easily separated, but this study demonstrated that white shark presence at two bathing beaches is strongly linked to water temperature, with increased probabilities of encountering sharks in warmer waters over the months September to May.

Water temperature is believed to be one of the most important environmental variables determining the distribution of sharks in coastal environments [21], [22], [23], [24], with water temperatures in False Bay playing an important role in determining seasonal fish assemblages and abundance in the bay’s surf-zones [25]. Water temperatures in False Bay vary seasonally from a mean winter temperature of 13.2°C to a mean summer temperature of 21.5°C [25]. This overall increase in water temperature is driven by the dominant onshore south-easterly wind in the summer months [26]. Within the spring and summer period, temporary upwelling events result in colder waters along the eastern and middle areas of False Bay and warmer waters in the shallower, especially northern areas of the Bay, which include Muizenberg and Fish Hoek [26]. The warmer water results in blooms of surf-zone diatoms, which are associated with an increase in abundance and diversity of teleosts and chondrichthyans [25], [27], which in turn are prey for white sharks [13], [28], [29]. These upwelling events are also responsible for an increase in beach-seine catches of teleosts and chondrichthyans along this stretch of coast [30], [31]. Since white sharks are capable of regulating their internal body temperature and tolerating a wide range of water temperatures [32], [33] it seems more likely that the result of an increase in sightings in warmer waters is related to the increase in availability of potential prey, rather than a physiological preference for warm water at such a narrow temperature range.

No direct relationship was found between sightings and wind direction and strength, which are known to influence water temperature [26]. The absence of an effect may be due to the lag effects of wind which were not incorporated into the model. Incorporating such complex interaction terms and time lags are difficult for the GAMM models to interpret, resulting in overly complex models with un-separable effects of each individual variable.

Lunar phase was significant and consistent for both Fish Hoek and Muizenberg, suggesting a periodic trend in the probability of shark sightings with the lowest frequency at full moon and the peak at new moon. The lunar (or tidal) cycle represents a predictable pattern which is often linked to animal behaviour such as aggregation, reproduction, and daily and seasonal migrations [34], [35]. Although the mechanism is unknown, new moon may provide favourable conditions for white sharks by offering an increased opportunity for feeding or a hunting advantage. White shark catches in bather protection gill nets set off Australian beaches also increased during new moon [36]. The authors proposed that new moon conditions either provided better hunting opportunities for white sharks, or the low light conditions meant that they were not able to detect the nets. New moon was also found to influence white shark presence at a seal colony in California where it was suggested that low light conditions favoured white sharks by camouflaging them from their northern elephant seal prey [37]. Our results provide evidence that the effect of new moon persists in daylight and may be linked to spring tides and associated activity of prey.

Time of day represented by morning and afternoon shifts was only significant for Fish Hoek beach, where the probability of sighting a shark was 1.6 times higher during the afternoon shift compared to the morning shift. This trend could either be attributed to better spotting conditions during the afternoon shifts at Fish Hoek e.g. reduced glare or may represent a behavioural trend unique to this area. Analysis of acoustic data from the same site as part of an ongoing telemetry study will reveal if this is indeed a behavioural trend specific to the area or not.

Our analysis determined that there has been a significant increase in the probability of sighting a white shark over the past three years at both beaches. Our data is not able to determine whether the increase is due to more sharks using these beaches, either through a change in distribution or a population increase, or whether it’s the same sharks using the beaches more regularly. Since white sharks have been protected by law for over two decades [13], [16], it is possible that an increase in sightings may be linked to a population increase. Regardless of the reasons for the increase, this trend, coupled with a growing human population, likely reflects an increase in risk to water users and highlights the need for an ongoing shark-attack mitigation strategy.

There is a more than four-fold variation in the probability of encountering a white shark along the bathing beaches across all environmental conditions in the months September to May which include the peak months for recreation. The pattern with respect to temperature and moon phase has been communicated by the Shark Spotters programme and City of Cape Town via a variety of media to increase the awareness of bathers to the onset of conditions that would suggest a higher risk of shark encounter. It is too early to assess whether the new information has altered bather behaviour. Further monitoring of the trends will allow for future investigations into changes in bather awareness and the probability of white shark encounters. Bather numbers are recorded hourly by Shark Spotters at all operational beaches, and by incorporating bather numbers into future models it will be possible to evaluate the success of reducing shark-attacks through increased bather awareness. The presence of beach seine-net fishing (i.e. trek netting) and other animal activity within the bay are additional variables considered for inclusion in the monitoring programme. Future research may expand to focus on trends revealed by the prey of white sharks in relation to water temperature and patterns of coastal marine activity around new moon. The greater our knowledge and understanding of the habitat use of large, predatory sharks (e.g. white sharks, bull sharks and tiger sharks) at popular bathing beaches, the higher the possibility of increasing water user safety and minimizing shark attacks and their subsequent negative conservation and economic repercussions.
